# Supplementary material for: RNA-Seq Reveals That Multiple Pathways Are Involved in Tuber Expansion in Tiger Nuts (Cyperus esculentus L.)
Source: Int J Mol Sci. 2024 May 7;25(10):5100. doi: 10.3390/ijms25105100 (PMC11121407; doi:10.3390/ijms25105100)
Supplement: Supplementary file 1 [file ijms-25-05100-s001.zip › Supplementary Figures.pdf]

Supplementary Figure Information

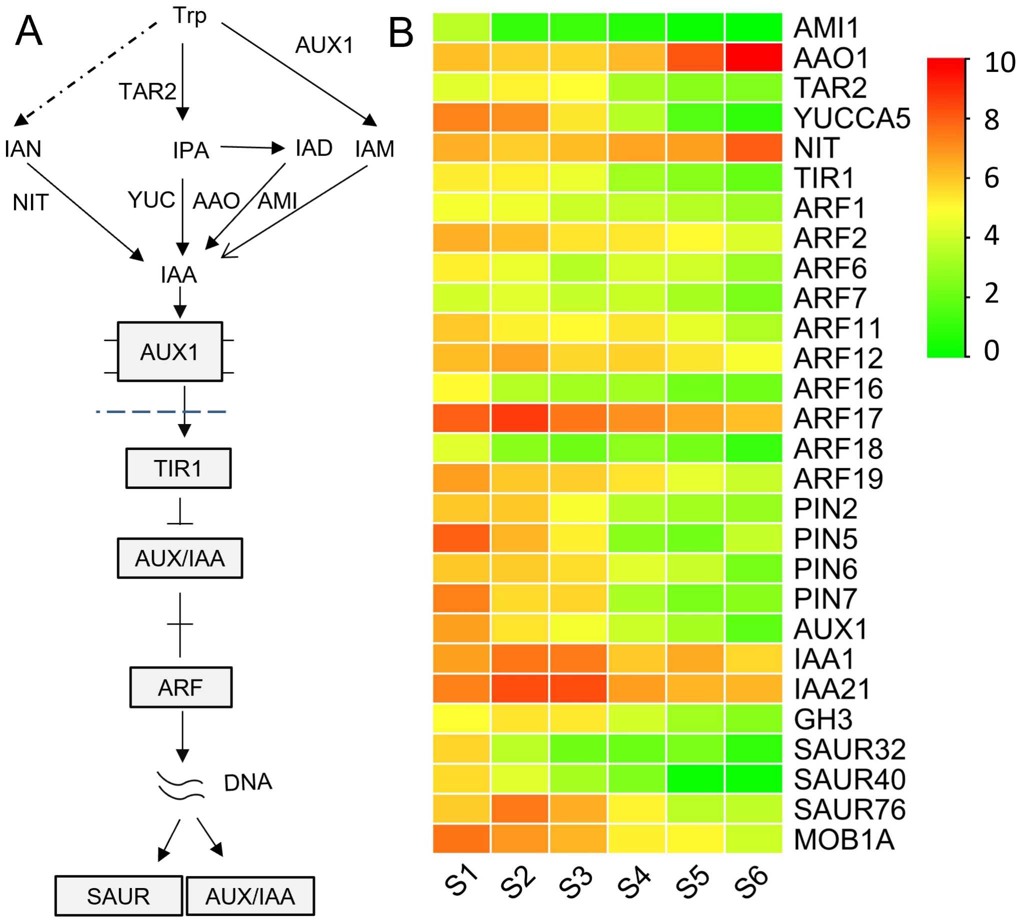

Figure S1. Analysis of DEGs related to IAA during tuber expansion. (A) IAA synthesis and signal transduction pathway; (B) Heatmap analysis of IAA synthesis and signal transduction related genes.

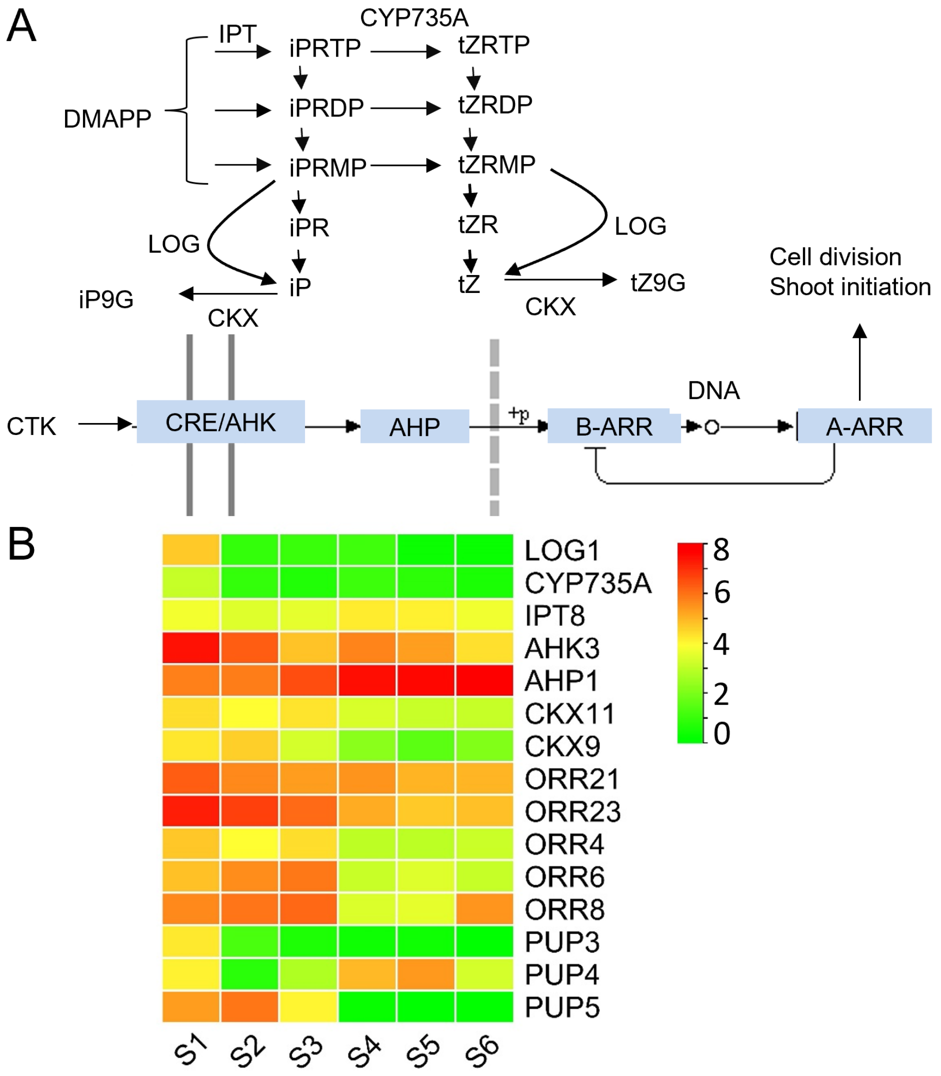

Figure S2. Analysis of DEGs related to cytokinin during tuber expansion. (A) Cytokinin synthesis and signal transduction pathway; (B) Heatmap analysis of cytokinin synthesis and signal transduction related genes.

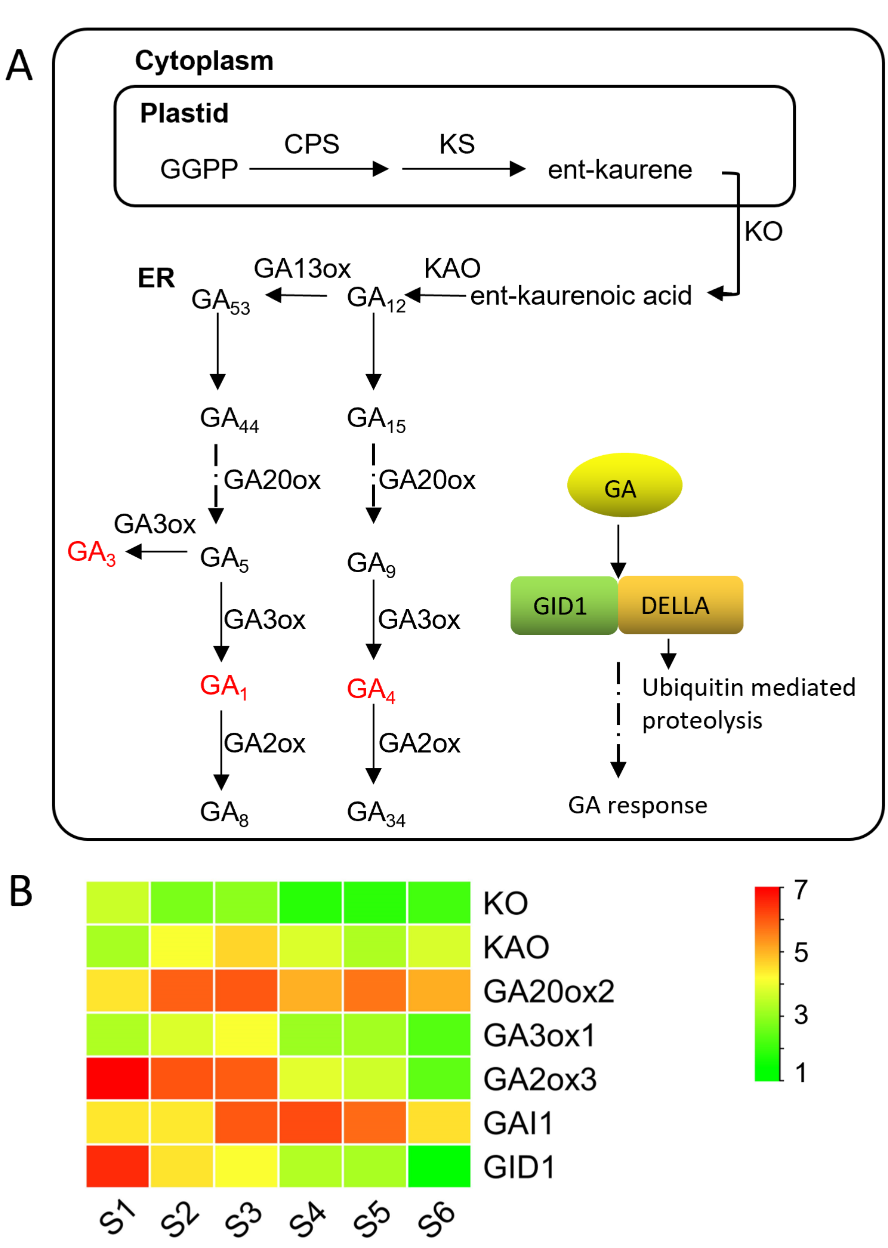

Figure S3. Analysis of DEGs related to gibberellin during tuber expansion. (A) Gibberellin synthesis and signal transduction pathway; (B) Heatmap analysis of gibberellin synthesis and signal transduction related genes.

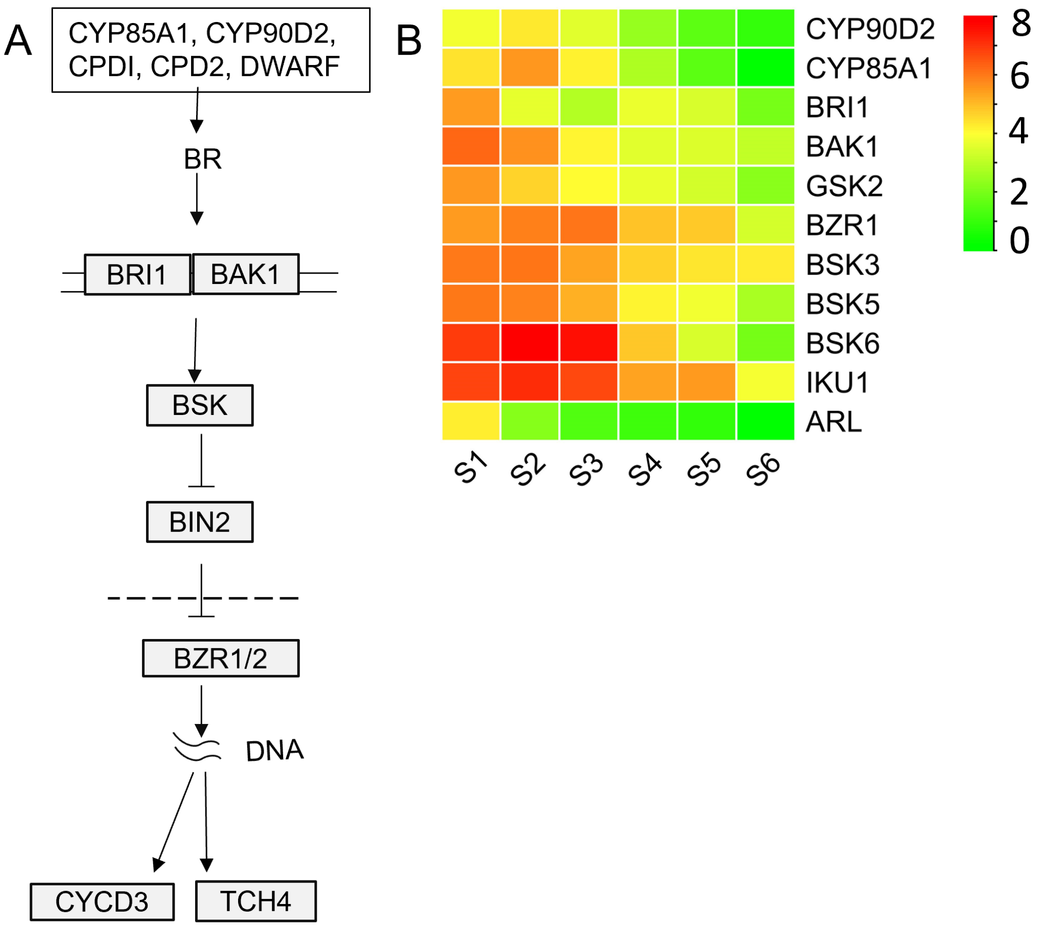

Figure S4. Analysis of DEGs related to brassinosteroid during tuber expansion. (A) Brassinosteroid synthesis and signal transduction pathway; (B) Heatmap analysis of brassinosteroid synthesis and signal transduction related genes.

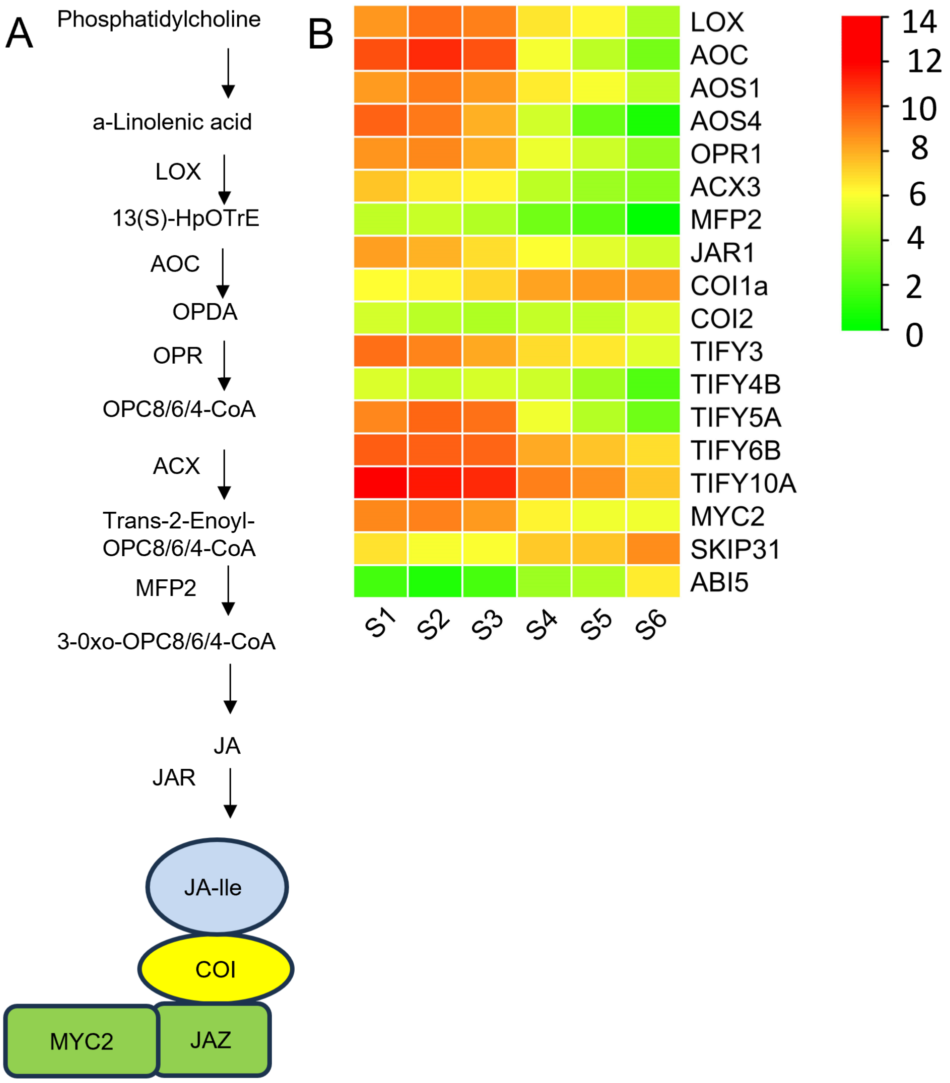

Figure S5. Analysis of DEGs related to jasmonic acid during tuber expansion. (A) Jasmonic acid synthesis and signal transduction pathway; (B) Heatmap analysis of jasmonic acid synthesis and signal transduction related genes.

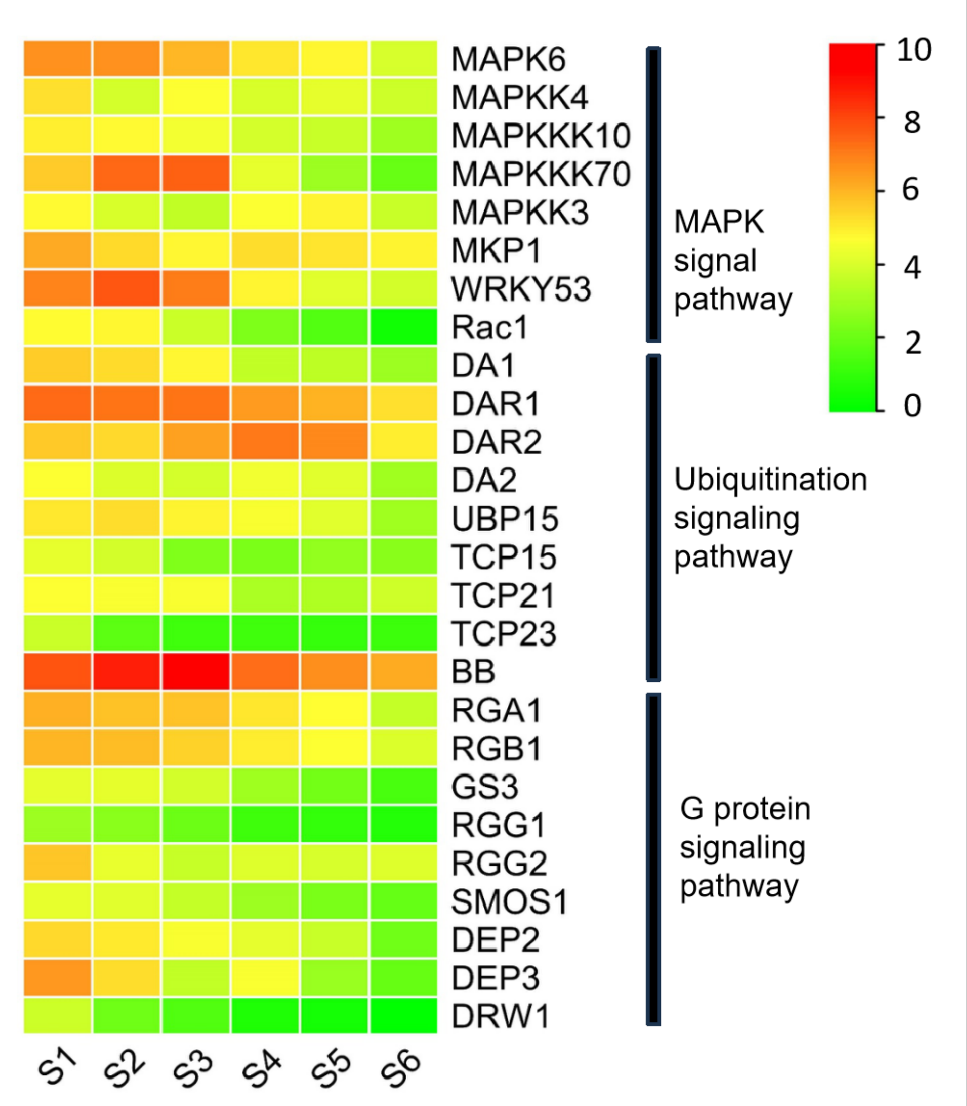

Figure S6. Heat map analysis of transcriptional regulation related DEGs.

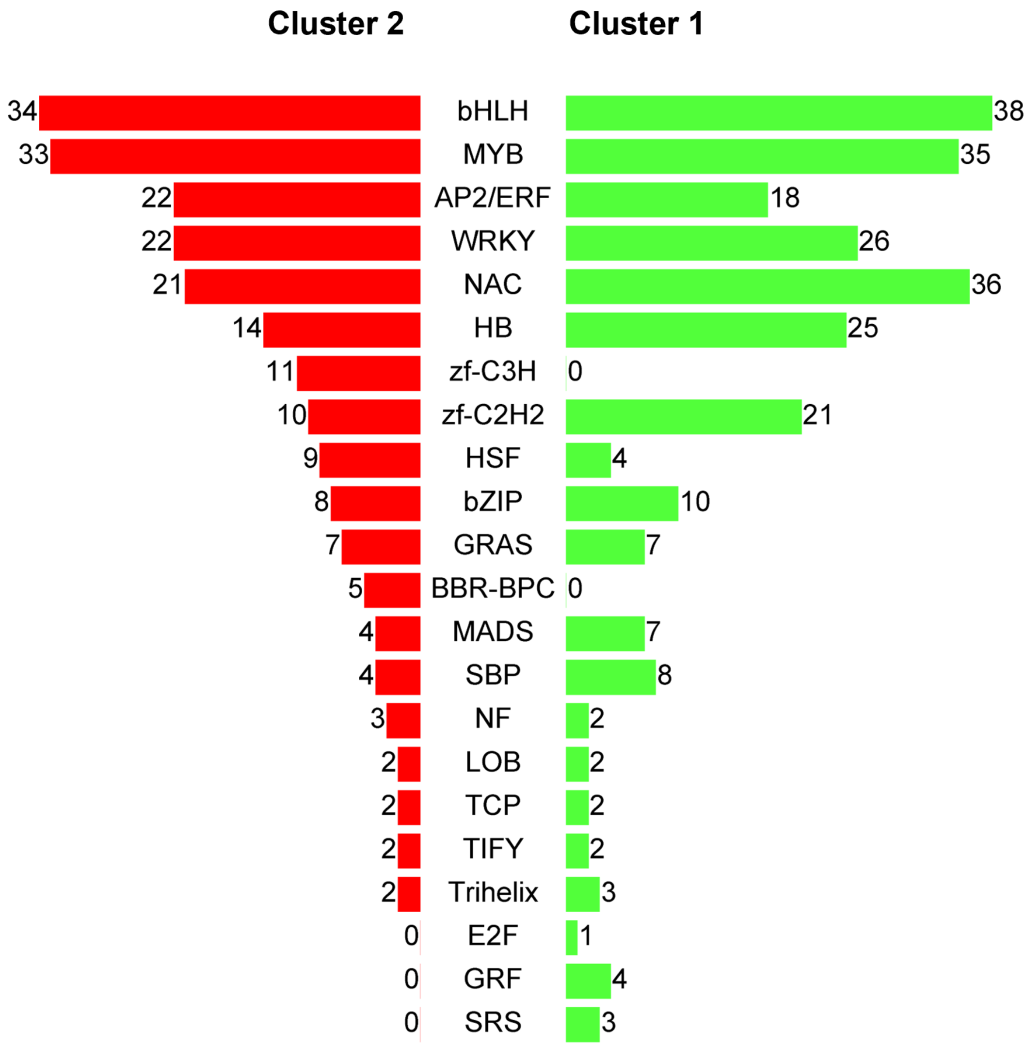

Figure S7. Differential expression of transcription factors during tuber expansion.
